# Neighborhood Preference of Amino Acids in Protein Structures and its Applications in Protein Structure Assessment

**DOI:** 10.1038/s41598-020-61205-w

**Published:** 2020-03-09

**Authors:** Siyuan Liu, Xilun Xiang, Xiang Gao, Haiguang Liu

**Affiliations:** 1 Complex Systems Division, Beijing Computational Science Research Center, Haidian, Beijing, 100193 China; 2 School of Software Engineering, University of Science and Technology of China, Hefei, Anhui 230026 China; 3 Physics Department, Beijing Normal University, Haidian, Beijing, 100875 China

**Keywords:** Protein structure predictions, Software, Statistical methods

## Abstract

Amino acids form protein 3D structures in unique manners such that the folded structure is stable and functional under physiological conditions. Non-specific and non-covalent interactions between amino acids exhibit neighborhood preferences. Based on structural information from the protein data bank, a statistical energy function was derived to quantify amino acid neighborhood preferences. The neighborhood of one amino acid is defined by its contacting residues, and the energy function is determined by the neighboring residue types and relative positions. The neighborhood preference of amino acids was exploited to facilitate structural quality assessment, which was implemented in the neighborhood preference program NEPRE. The source codes are available via https://github.com/LiuLab-CSRC/NePre.

## Introduction

Despite the advances in protein structure determination methods, the discovery rate of new proteins greatly exceeds the rate of experimental structure determination. New proteins can be discovered by high-throughput genome sequencing using sophisticated genome analysis tools^1–3^. In contrast, the protein structure determination requires complicated procedures to obtain high-quality protein samples that produce sufficiently good experimental signals. For example, the target protein must have a reasonably high expression rate to obtain enough sample, after which the protein is purified, followed by the optimization of crystallization cocktail recipes to yield high-quality crystals for X-ray crystallography^4,5^. Alternatively, the molecules must be labeled using isotopes for specific atoms for nuclear magnetic resonance^6,7^. Recent breakthroughs in cryogenic electron microscopy methods have indicated that structure determination can be achieved without tedious crystallization or isotopic labelling^8^. However, the technology is not yet highly automated and requires extensive computational analysis of a large volume of data for each structure. Limitations to experimental structure determination of protein molecules necessitate the development of methods for protein structure prediction using computational modeling approaches.

Protein structure prediction has a long history marked by prediction contests, such as the Critical Assessment of protein Structure Prediction (CASP), which was first organized in 1994^9,10^. Structure prediction has achieved successes in many cases and is used in numerous applications^11^. Particularly, predicted structures can be combined with experimental data to comprehensively understand the structure and function of molecules^12–16^. In many cases, it is difficult to determine a high-quality structure based solely on experimental information. Hybrid methods that integrate structure prediction results and experimental data are promising for exploiting information from both experimental data and computational modeling or predictions^16–18^. For a structure prediction method to be successful, it must have two components: (1) an algorithm to generate a structure ensemble that includes good models, i.e., at least some models in the ensemble are similar to the correct structure (or the native structure); and (2) a scoring function that can rank the generated structures, so that the good models can be identified. Scoring functions either can guide the sampling of protein conformations to improve sampling efficiency, or can be used independently to assess model quality. The structure ensemble of a protein, also referred to as a decoy set, can be generated using several computational methods. The mainstream methods include homology modeling^19^, structure threading^20,21^, and segment assembly^22–24^. Advanced sampling algorithms can be applied to ensure the diversity of conformations in decoy sets to increase the chance of sampling the structure with the lowest energy^24^. In this study, we focused on the scoring function used to assess the quality and correctness of each generated model.

There are two types of scoring functions. One is based on physiochemical principles—force fields in molecular modeling, such as Amber or Charmm for atomic models^25,26^ and Martini or UNIRES for coarse-grained models^27–29^. The other type can be classified as empirical energy functions based on statistical knowledge of experimentally determined structures. There has been tremendous success in applying empirical energy functions to analyze protein structures. One famous example is the protein main chain dihedral angle distributions, known as the Ramachandran plot^30^, which is widely used for protein structure validation^31–33^. Representative developments in empirical energy functions include PROSA, DFIRE, DOPE, RW, RWplus, and GOAP^34–38^. Orientation-dependent force fields were used to demonstrate the importance of incorporating the relative positions of amino acids^39,40^. Recent development in machine learning have also led to new frameworks for interaction energy development^41–46^. Inspired by these pioneering works, we developed a new energy function that describes amino acid neighborhood preferences. For each of the 20 natural amino acids, the neighboring amino acid was analyzed in detail with a focus on orientation preferences, described using the polar angle parameters. Distance-dependent energy functions have been well-studied and incorporated into existing methods, thus we focused on orientation-dependent energy functions in this study. Specifically, the preference was determined using 400 (20 × 20) matrices that describe the relative positioning (i.e., orientation) of any two amino acids. For any two amino acids, the probabilities of being neighbors and their relative positions were extracted from a high-resolution structure dataset. The probability distributions were converted to energy functions using the Boltzmann relation, and these energy functions were used to assess the quality of the decoy structures. Based on the results and the performance comparison with several other methods, we found that the neighborhood preference (NEPRE) program is effective for ranking decoy structures and quantifying the correctness of protein structures.

## Methods

The native state structures of proteins are stabilized mostly by the interactions between atoms that are not covalently bonded, mainly including electrostatic and van der Waals interactions. Although these interactions are nonspecific, each amino acid is found to have preferences for its neighboring amino acid types, particularly its nearest neighbors. Furthermore, the relative positions of neighboring amino acids are critical for their packing in protein 3D structures. With this in mind, we carried out detailed statistical analysis on the neighborhood preference of each type of amino acid. First, a local coordinate system was established for each amino acid to describe its neighboring amino acid positions; the neighboring residues were mapped to the spherical coordinates defined around the amino acid of interest; these analyses were repeated for every amino acids in the protein structure to obtain statistics for the overall neighborhood preference. The final statistics were obtained from a non-redundant dataset composed of 14,647 PDB structures, with a sequence similarity cutoff at BLAST p-value of 10^−7^ (https://www.ncbi.nlm.nih.gov/Structure/VAST/nrpdb.html)^47^. All chains in the PDB were compared using the BLAST algorithm, followed by clustering using a single-linkage clustering procedure. At a p-value cutoff of 10^−7^, structures with better sequence completeness and higher resolutions were selected to represent each group. The structures in this dataset were restricted to single-chain proteins to derive the intra-chain neighborhood preferences.

### Local coordinate system for each amino acid

**Figure 1:**
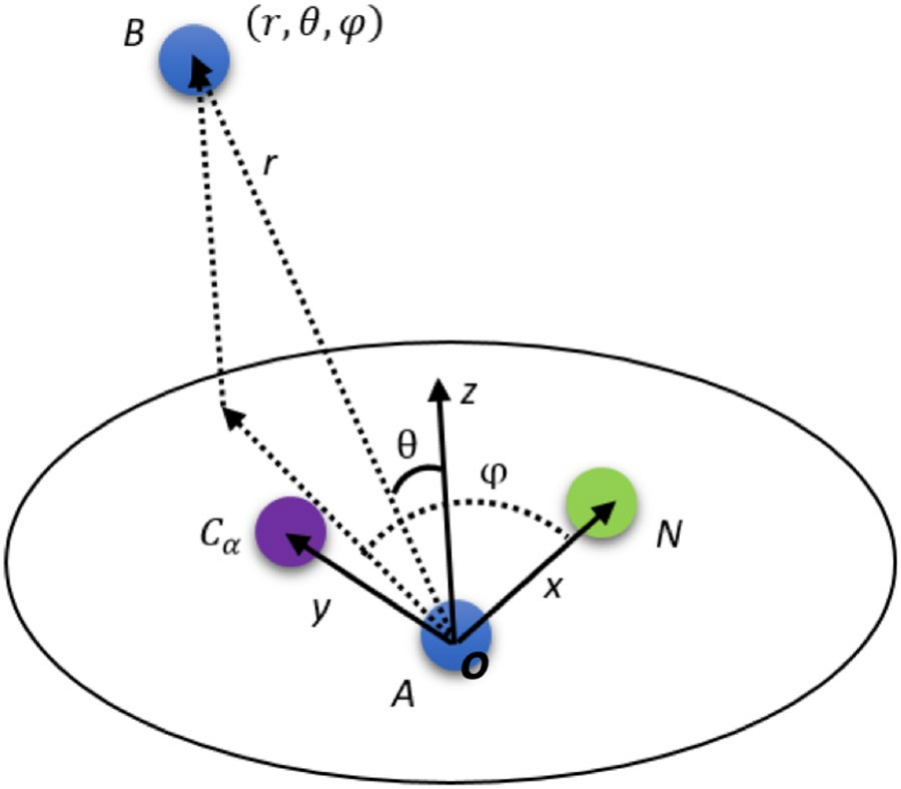
Schematic drawing of two neighboring amino acids (A and B). Location of amino acid B (represented using its geometry center) is shown in the coordinate system of amino acid A, defined with the positions of ***Cα, N*** atoms, and its geometric center (***o***).

The local coordinate system was defined using the main chain atoms of each amino acid, as described previously^48^. This is the foundation of neighborhood analysis for each amino acid. Briefly, the geometry center of each amino acid was calculated as the average position of the associated atoms, then the X-Y plane was defined using the geometry center (g_c,_ labeled as “***o***” in Fig. 1) of the amino acid, nitrogen atom (***N***), and carboxyl carbon atom (***Cα***). The geometry center, ***o***, is set to be the origin point of the local coordinate system for the amino acid (see Fig. 1). The positive x-direction is defined as ***o → N***, and then the positive y-direction can be defined in the X-Y plane such that the carboxyl atom ***Cα*** has a positive y coordinate. The z-direction is subsequently defined using the right-hand rule (Fig. 1).

The neighboring amino acids were selected based on the distances between the centers of the corresponding amino acid side chains. If the distance was within a given cutoff value (r_c_), they were considered as neighbors. Once the neighborhood was defined, statistical analysis was carried out for the amino acids located within the cutoff distance. We used two approaches for the distance cutoff: a universal fixed cutoff for all amino acids or the cutoffs specific to the types of neighboring amino acids.

For the case of a universal fixed cutoff, algorithm performance was tested using various cutoff values, with r_c_ between 4 and 10 Å. For the type-dependent case, the cutoff was determined by summing the radii of two neighboring amino acids. The radii of 20 amino acids were obtained from the same non-redundant structure dataset.

### Statistical model for amino acid contacts in protein molecules

The distribution function is related to energy via the quasi-Boltzmann’s relation^49^; particularly, energy can be expressed as:

1 $${\rm{E}}=-kTlog\frac{{p}_{obs}}{{p}_{exp}}$$

where $${p}_{obs}$$ and $${p}_{exp}$$ are the observed and expected probabilities in the subspace specified with parameters of interest. In NEPRE, $${p}_{obs}$$ and $${p}_{exp}$$ are specified with five parameters, $$(i,j,r,\theta ,\varphi )$$, where ($$i,j)$$ are the types of amino acids, and $$(r,\theta ,\varphi )$$ represent the relative coordinate parameters of the latter (*j*) in the former (*i*) amino acid’s local coordinate. To simplify the representations, the geometric center of each neighboring amino acid (***B*** in Fig. 1) was used to describe its location in the local coordinates of centered amino acid (***A*** in Fig. 1). From the structure database, the observation of amino acid type *j* in the neighborhood of amino acid type *i* is expressed as $${P}_{obs}(i,j,r,\theta ,\varphi )$$, with

2 $${P}_{obs}(i,j,r,\theta ,\varphi )=\frac{{N}_{ij}(r,\theta ,\varphi )}{{\sum }_{i,j}\,{N}_{ij}}=\frac{{N}_{ij}}{{\sum }_{i,j}\,{N}_{ij}}\ast \frac{{N}_{ij}(r,\theta ,\varphi )}{{N}_{ij}}={p}_{ij}\ast {p}_{ij}(r,\theta ,\varphi )$$

The expected values of the distribution of various amino acids are expressed as:

3 $${P}_{exp}(i,j,r,\theta ,\varphi )={p}_{i}\ast {p}_{j}\ast {r}^{2}sin\theta \varDelta r\varDelta \theta \varDelta \varphi $$

According to the above derivation, we obtained:

4 $$E(i,j,r,\theta ,\varphi )=-kTlog\frac{{P}_{obs}(i,j,r,\theta ,\varphi )}{{P}_{exp}(i,j,r,\theta ,\varphi )}=-kT\,\log (\frac{{P}_{ij}}{{P}_{i}{P}_{j}}\frac{{P}_{ij}(r,\theta ,\varphi )}{{r}^{2}sin\theta \varDelta r\varDelta \theta \varDelta \varphi \,})$$

where $k$ is the Boltzmann constant, $T$ is the temperature factor, and $(r,\theta ,\varphi )$ is the spherical coordinate of amino acid $j$ in the local coordinate system of amino acid $i$. Because absolute energy values are not required for ranking, we set *k*T = 1 in the program implementation. If necessary, the energy can be multiplied by *k*T to obtain physically meaningful values.

For a protein with *M* amino acids, the total energy ***E*** can be expressed as:

5 $$E=\sum _{m=1,M}\sum _{n\in \{n\}}E(t(m),t(n),r,\theta ,\varphi )$$

where *E*(…) is the pairwise statistical energy described in Eq. (4), *m* is the index of the amino acid, {*n*} is the indices of neighboring amino acid within the given distance cutoff of the amino acid *m*, and t(x) is the function that maps the amino acid indices to their types.

In the NEPRE implementation, the radial distance *r* was integrated from 0 to the distance cutoff ${r}_{c}$. Therefore, the statistics were simplified to the distributions in the sections specified by the angle parameters ($\theta ,\varphi )$ within the contacting sphere. A regular grid system was used to divide the sphere into 20 × 20 regions (see Discussion section for other gridding schemes and the performance comparison in Figures S2 & S3), with angular intervals $\varDelta \theta =\frac{\pi }{20}$ and $\varDelta \varphi =\frac{2\pi }{20}$ (because the range for $\theta \,is\,[0,\pi )\,{\rm{and}}\,{\rm{for}}\,\varphi \,{\rm{is}}\,[0,2\pi ))$. The unequal volume divisions were corrected by using the appropriate probability in the respective volume (see Eq. 3).

### Testing decoy datasets

The performance of the algorithms was tested with publicly available decoy datasets. After a careful literature survey, we identified five published datasets (390 decoy sets in total): the I-Tasser dataset, denoted as I-Tasser(a), and four datasets generated using the 3DRobot programs, including I-Tasser(b), 3DRobot, Rosetta, and Modeller. Information about the datasets is summarized in Table 1. The I-Tasser(a) dataset was generated using the original I-Tasser protocol, where Monte Carlo simulation was used to assemble the structure scaffolds that can be aligned to models in the database^36^. The 3DRobot algorithm extended the I-Tasser method to allow structure sampling without restraints on the fragments, thus generating more diverse conformations for scoring function benchmarking^50^.

**Table 1:** Summary of the five datasets.

Dataset Name	Protein size	No. of protein decoy sets	Number of structures in each decoy set	References
I-TASSER (a)	47–118 aa	56	400	^36^
I-TASSER (b)	47–118 aa	56	400	^36,50^
3DRobot	80–250 aa	200 (48 α-, 40 β-, and 112 α/β-single-domain proteins)	300	^50^
Rosetta	50–146 aa	58	100	^50,55^
Modeller	81–340 aa	20	200	^50,56^

The decoy structures were evaluated using the proposed NEPRE scoring function. Two metrics were used to characterize the ranking: (1) the success rates in identifying the native structures (or the most native-like structures); and (2) the Pearson correlation between the energy and root-mean-square-deviation (RMSD) with respect to the native structures.

## Results

### Type specific neighboring preferences for amino acids

**Figure 2:**
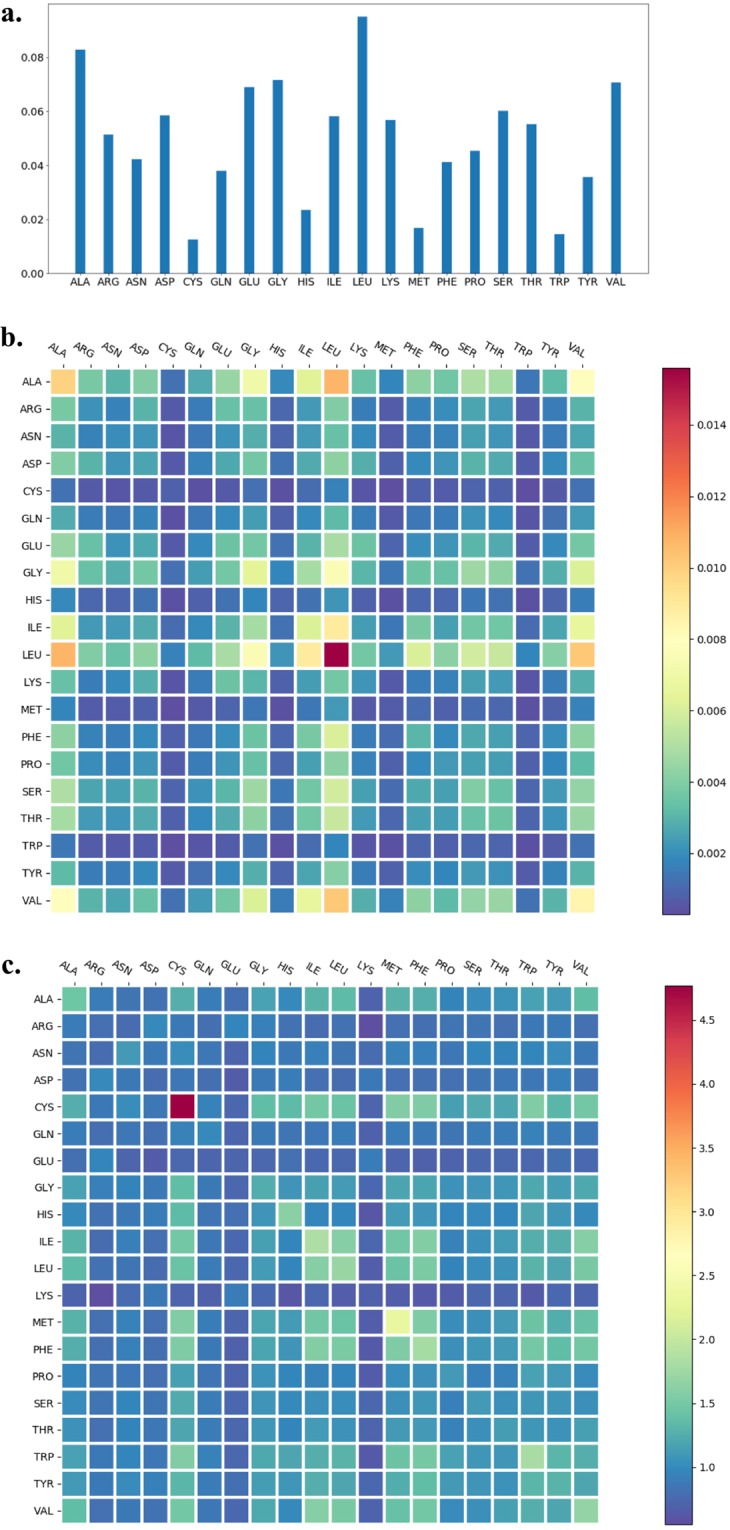
Probability of observing amino acids and amino acid neighbors. (**a**) Amino acid abundance (normalized) in the protein dataset; (**b**) Probability of amino acid pairs in the spatial neighborhood; (**c**) Observed to expected ratios for neighboring amino acids.

The 20 natural amino acids appear in protein molecules with different abundances. The probability of finding a specific type of amino acid in the non-redundant dataset is summarized in Fig. 2a, showing that hydrophobic amino acids, such as leucine, alanine, and valine, appear in protein molecules more frequently than the other amino acids. The probabilities of observing two spatially neighboring amino acids for the 20 × 20 pairs were shown in Fig. 2b for the case with a distance cutoff value of 6.0 Å. The neighborhood preference was quantified using the “observed to expected ratio”, o/e, defined as $\frac{p(i,j)}{p(i)p(j)}$, shown in Fig. 2c. Certain amino acid types exhibited strong preferences for their neighbors, for example, cysteine strongly prefers another cysteine in its neighborhood (consistent with the observation of disulfide bonds). The type preferences, together with the orientation preferences described in the following, are useful for quantifying the packing of amino acids in protein structures.

### Position preferences of amino acids in the neighborhood of each amino acid

**Figure 3:**
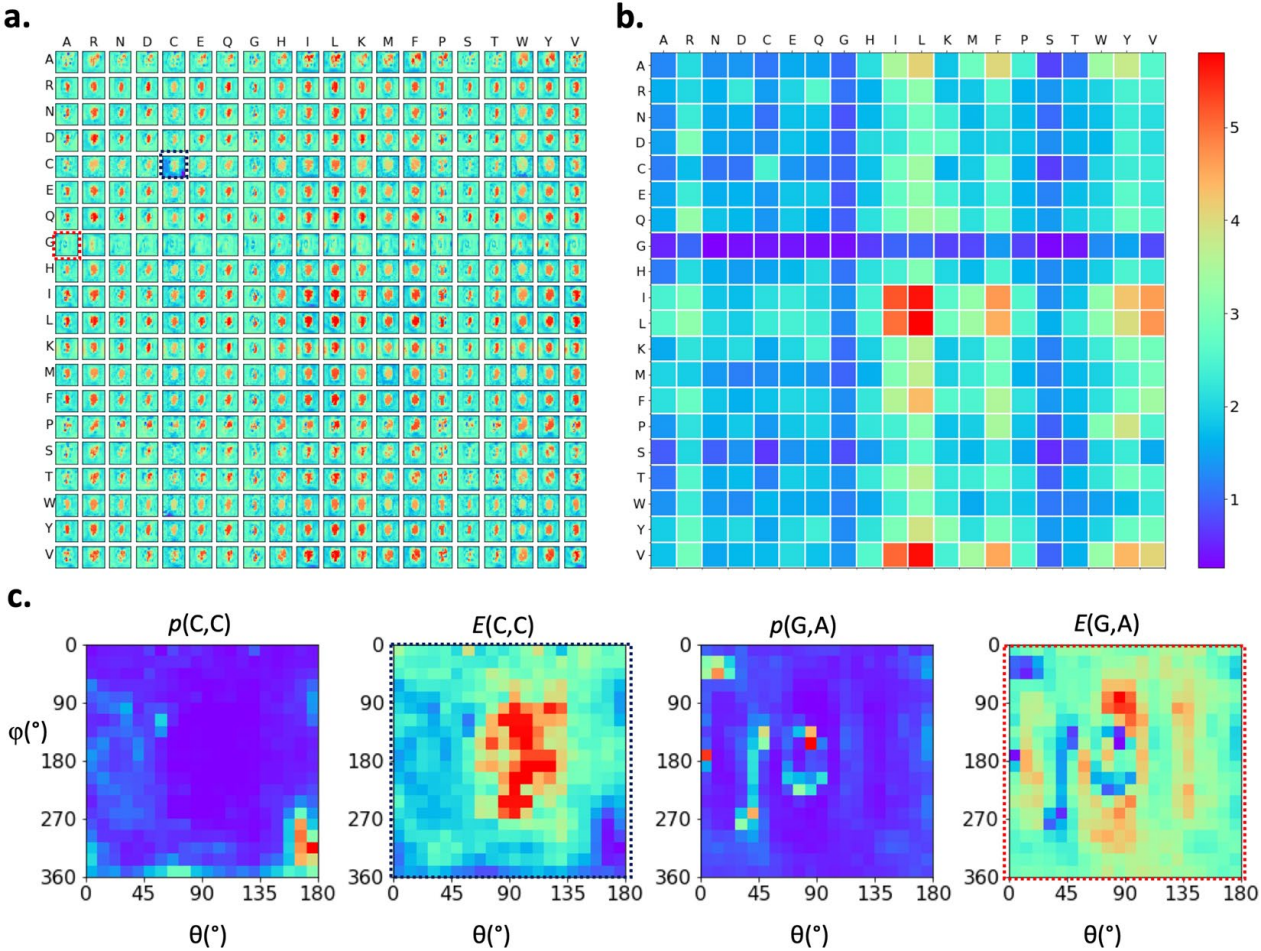
Distributions of amino acid in the neighborhood of another amino acid. (**a**) Position-dependent energy functions of all 20 × 20 pairs of amino acids. Each row shows the neighborhoods centered at a specific amino acid type; and each entry in a row is the interaction energy between a neighboring amino acid and the centered amino acid. (**b**) Energy variances for each amino acid pair shown in (**a**). (**c**) Two representative amino acid neighboring preferences in the forms of probability distributions and energy functions, showing position preferences: cysteine in the neighborhood of cysteine (*p*(C,C) and *E*(C,C) shown on the left-hand side) and alanine in the neighborhood of glycine (*p*(G,A) and *E*(G,A) on the right). The corresponding energy functions are enclosed with dashed lines in (**a**).

The amino acids interact with each other in their preferred positions, as revealed by the non-uniform distribution of one amino acid within the neighborhood of another amino acid (i.e., within the sphere centered at one amino acid). This provides additional information to the preferred types discussed in the previous section. In Fig. 3a, the pairwise energy function in the angle space (θ,φ) in the spherical coordinate are shown for all 20 × 20 amino acid pairs (see Eq. 4). Figure 3b shows the energy variances for each amino acid pair, corresponding to the distributions shown in Fig. 3a. Larger fluctuations (towards red colors) indicate stronger position preferences and vice versa. The probability distributions (*p*) and energies (*E*) of two examples (CYS around CYS, ALA around GLY) are shown in Fig. 3c. Cysteines showed distinguishable preferences for their neighboring cysteine residues, mainly concentrated in the red-colored region around (162°, 270°) in the left figure of Fig. 3c, corresponding to the conformation of disulfide bonds. For the case of alanine around glycine (two figures on the right-hand side of Fig. 3c), the probability distribution was less polarized, indicating that the interactions between alanine and glycine do not have particular strongly preferred positions. Next to the probability distributions, the corresponding pairwise interaction energy functions (Eq. 4) in the angle space are shown.

### NEPRE performance of selecting native structures

As described in the Methods section, the NEPRE program has two implementations depending on the choice of neighborhood cutoff values. One implementation utilizes a fixed cutoff value for all 20 types of amino acids, hereafter named as NEPRE-F (fixed cutoff); the second implementation uses cutoff values depending on the neighboring amino acid radii, named as NEPRE-R (radius-dependent cutoff).

The cutoff value is critical for the neighborhood boundary in the case of NEPRE-F; hence, we tested the scoring function at various cutoff values from 4 to 10 Å for the five datasets described in the Methods section (Table 1). The success rates for identifying the native structure from each decoy set are summarized in Table 2.

**Table 2:** Number of success cases with different neighborhood distance cutoffs.

Cutoff	I-TASSER (a)	3DRobot	I-TASSER (b)	ROSETTA	Modeller
4 Å	9/56	40/200	5/56	7/58	9/20
5 Å	44/56	126/200	18/56	25/58	12/20
**6 Å**	**48/56**	**149/200**	**22/56**	**34/58**	**13/20**
7 Å	49/56	140/200	20/56	25/58	14/20
8 Å	49/56	119/200	19/56	18/58	13/20
9 Å	49/56	104/200	20/56	14/58	11/20
10 Å	49/56	89/200	20/56	9/58	11/20

**Figure 4:**
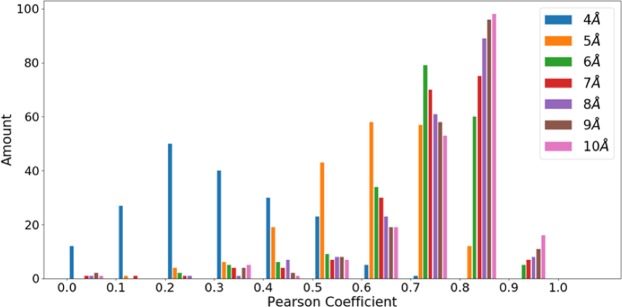
Overall ranking quality measured using Pearson correlation coefficients. The distributions of Pearson correlation coefficients are shown at distance cutoffs from 4 Å to 10 Å.

The overall performance of NEPRE-F was the best when the cutoff = 6 Å, where the native structures were scored as those with the lowest energy in 266 of 390 decoy sets. The second-best cutoff value was 7 Å, with 248 native structures identified, followed by the case with cutoff = 5 Å, identifying 225 native structures. Based on this criterion, a cutoff = 6 Å is a good choice for selecting native structures from decoys and was used as the default value for structure assessment using NEPRE-F. We also analyzed the correlation between the scoring function and structural difference with respect to the native state in each decoy (quantified using the RMSD with respect to the native structure, see Figure S4 for an example). Interestingly, we found that the correlation increased as the cutoff increased, with the cutoff = 10 Å giving the best Pearson correlation coefficients (Fig. 4). Considering that the ultimate goal of the scoring function is to select the native (or near native) structures from decoys, we used a cutoff = 6 Å as the default parameter in the following analysis.

For the case of NEPRE-R, the radii for each type of amino acid were extracted from the non-redundant dataset. The distributions of radii for 20 amino acids are shown in Figure S1 and the mean values are summarized in Table 3. These values were used to determine the cutoff values in the neighborhood statistics for specific amino acid types. For neighborhood analysis, the associated energy function was derived in the same manner as in the case of NEPRE-F.

**Table 3:** Amino acid radii extracted from the database.

Amino acid type	Radius (Å)	Amino acid type	Radius (Å)
ALA	3.20	LEU	4.24
ARG	5.60	LYS	5.02
ASN	4.04	MET	4.47
ASP	4.04	PHE	4.99
CYS	3.65	PRO	3.61
GLN	4.64	SER	3.39
GLU	4.63	THR	3.56
GLY	1.72	TRP	5.38
HIS	4.73	TYR	5.36
ILE	3.94	VAL	3.55

### Performance of native structure selection compared to other methods

Using the five decoy sets, we evaluated the performance of several widely used methods with statistical potentials (DFIRE2, DOPE, GOAP, RW, and RWplus), whose executable programs can be obtained from internet. The success rates in identifying the native structure or narrowing down the native structures to a smaller number of candidates (5 or 10) were used to assess performance. As shown in Table 4, NEPRE-R and NEPRE-F showed advantages in recognizing the native structures (represented as the number of TOP1 selected by each scoring function). If the success rates for the native structure included 5 or 10 structures with the lowest energies (indicated with TOP5 or TOP10 in Table 4), rather than the stringent requirement to be the lowest energy structure, we observed that the NEPRE algorithm performed well in all decoy sets, and NEPRE-F yielded better results than NEPRE-R. The scoring function with the best performance in each dataset is highlighted in bold font in Table 4.

**Table 4:** Performance comparison of different potentials.

		DFIRE2	DOPE	RW	RWplus	GOAP	NEPRE-R	NEPRE-F
I-TASSER(a)	Top1^§^	53	48	54	56^*^	3	50	48
(56)^#^	Top5	55	48	55	**56**	14	50	48
	Top10	55	49	55	**56**	17	53	50
I-TASSER(b)	Top1	0	11	0	0	3	20	**22**
(56)	Top5	2	25	1	2	3	27	**28**
	Top10	2	29	4	4	3	29	**33**
Rosetta	Top1	0	7	0	0	1	26	**34**
(58)	Top5	2	31	2	2	7	45	**49**
	Top10	5	43	8	8	7	51	**53**
Modeller	Top1	2	6	2	2	1	**14**	13
(20)	Top5	4	8	4	4	5	**16**	**16**
	Top10	6	10	6	7	6	17	**18**
3DRobot	Top1	38	63	0	0	4	129	**149**
(200)	Top5	49	141	5	8	13	160	**173**
	Top10	60	165	9	10	15	176	**183**

^#^Number of proteins.

^§^The number of cases whose native structures are in the *n* best models selected based on scoring functions.

^*^Numbers in bold font indicate the best scoring function for that assessment.

Considering that native structures may not be among the decoy set sampled using the model generation programs, we tested the NEPRE’s performance on selecting the decoy structures most similar to the corresponding native structures (i.e., those with the smallest RMSD with respect to the native structures). Interestingly, we found that the performance was not as good in identifying native structures. For example, for the Modeller dataset, NEPRE-R and NEPRE-F identified the native structures in 14 and 13 of 20 cases, respectively, which was much better than for all other methods (see Table 4). In identification of the best decoy structure, the numbers were 6 and 5 (of 20 cases) for NEPRE-R and NEPRE-F, respectively. In contrast, DOPE performed the best in identifying the best decoy structures, succeeding in 8 cases for the Modeller dataset. The other scoring functions showed similar performance as the NEPRE methods. The detailed comparison results indicate that the NEPRE algorithm effectively identified the native (or near native) structures. If no structures are ‘native-like’ in a decoy set, then the NEPRE may not select the structure with the smallest RMSD. However, the positive correlation between the energy and RMSD is still valid in most cases (see Fig. 4).

### Performance on CASP12 decoy datasets

**Figure 5:**
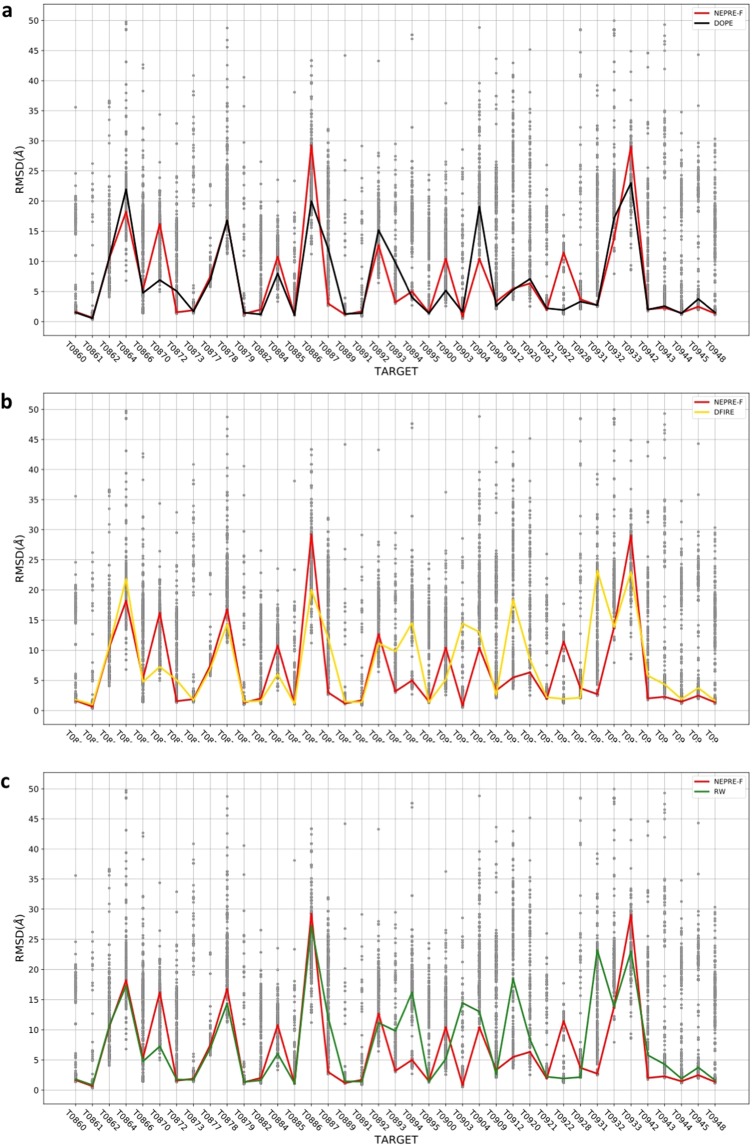
Performance of NEPRE-F in ranking the CASP12 decoy sets. The lines indicate the position of identified decoy structures with the lowest energies. The performances against three other scoring functions are compared: (**a**) DOPE; (**b**) DFIRE; (**c**) RW.

Among the CASP12 targets, we selected a subset of proteins whose native structures have been determined by X-ray crystallography. This dataset contains 39 decoys composed of predicted models submitted by participants of the CASP12. The results of best decoy structure identification are summarized in Fig. 5, in which the performance of NEPRE-F is compared to that of the DOPE, DFIRE, GOAP, and RW programs (the results of the RWplus were very similar to those of RW; see Figure S5). The DOPE program performed the best in identifying the decoy structure with the smallest RMSD with respect to the native structure, which is consistent with the testing results using the 390 generated decoy sets as summarized in the previous section. NEPRE-F outperformed DFIRE and RW in the CASP12 dataset. NEPRE-F identified decoy structures with RMSD < 3 Å in 18 of 39 cases. For the other methods, the number of successful cases was 18, 15, and 16 for DOPE, DFIRE, and RW, respectively. Furthermore, for decoy sets containing structures with RMSD < 5 Å, NEPRE-F successfully identified at least one of these good structures in 26 out of 31 targets (for all participants, eight targets were too difficult to predict any structure with RMSD < 5 Å; see Fig. 5). Thus, NEPRE-F made good predictions in 26 decoy sets by successfully identifying structures with RMSD < 5 Å and failed in five sets (31 decoy sets in total). In CASP performance evaluation, GDT_TS is often used to measure the similarity of the predicted model to the experimentally determined model^51^. The performance of NEPRE was compared to that of the other five potential energies; the results indicate that DOPE and GOAP performed best in selecting models with the best GDT_TS scores, and the performance of NEPRE was very similar to that of the other three methods (see Figure S7).

## Discussion and Conclusion

In protein structures, amino acids exhibit preferences for their neighboring amino acids, in both amino acid types and relative positions. This property was systematically studied using experimentally determined structures. Based on the results of neighborhood preference, we developed a new algorithm, NEPRE, which is generally applicable for structure assessment of single-chain proteins. We tested this algorithm using five published decoy datasets (390 decoy sets in total) and a new dataset composed of 39 decoy sets formed the predicted models in CASP12. The performance of the NEPRE algorithm showed potentiality for structure prediction. The execution time was 3–4 s for proteins in the tested decoy sets, including PDB file parsing and energy calculation. Therefore, it is feasible to integrate the NEPRE algorithm in model generation programs to guide the sampling of desired structure ensembles. In addition, we have tried to compare the preferences of amino acids in single chains with that laying in the interfaces of protein complexes. Using a dataset extracted from the PISA database^52^, we analyzed amino acid packing at the complex interfaces and found that the neighborhood preferences were similar to the results presented in this study in most cases (see Figure S8), with pronounced differences observed for alanine, cysteine, glycine, and valine. This indicates that the interface specificity of amino acid neighboring preferences are different. The application of NEPRE at the interfaces and detailed comparison of the preferences will be reported in a separate study.

There are two major considerations when discretizing the angle space into the regular grid: (1) the potential energy profile needs to be fine enough to accurately describe the neighborhood (i.e., amino acid packing preferences); and (2) the discretized region is large enough, so that the sampling extracted from the non-redundant database is sufficient for statistical significance. For the first consideration, we evaluated the performance of NEPRE using four discretization schemes, with 15, 20, 25, and 30 discrete sections. The results showed that finer discretization revealed more features, whereas a larger number of discretized regions was under-sampled. As a compromise, we chose N = 20 to balance the two considerations. Furthermore, using the decoys in the Modeller dataset, we compared the ranking performance and found that the performance in the scenario with N = 20 was nearly the same as that in finer discretization cases (see Supplementary materials). It is worthwhile to point out that discretization in angle parameter space can be improved to yield regions with nearly uniform volumes by using algorithms such as Fibonacci lattice or HEALPix^53,54^.

The NEPRE algorithm was implemented in two forms depending on the cutoff value defining the neighborhood. The results showed that a cutoff = 6 Å is a good choice for all amino acids types. This performance of NEPRE-F was further validated using CASP12 decoy sets that were not used to determine the optimal distance cutoff values. The performance of NEPRE-F is better than that of NEPRE-R, whose distance cutoff values depend on the neighboring amino acid sizes. Intuitively, type dependent cutoff values should describe more precise interactions between amino acids, and therefore should lead to better performance. While the exact causes for NEPRE-R’s inferior performance compared to its peer NEPRE-F (with cutoff = 6 Å) remain unclear, there are several possibilities. The radius values for each amino acid were obtained from the statistics in the protein structures, and the average values may not accurately reflect the neighboring interactions with other amino acids. For example, cysteine and serine each has two peak values, and using a single average value may result in misrepresentation of the neighborhood (see Supplementary materials). A universal fixed cutoff may define a neighborhood that is more suitable for the residue-level scoring function, as the distances between amino acids were measured using distances between their geometry centers.

The NEPRE algorithm performed well in recognizing the native structures in all five decoy datasets. In contrast, most other methods showed variations in their performances across the datasets. For example, the other five methods successfully recognized most of the native structures for dataset I-TASSER(a), but the success rates were lower for other four datasets. This reflects the challenges raised by the four datasets generated with the 3DRobot algorithm, which enhances the conformational diversity. If the native structure is absent (such as in the case of CASP competitions), the performance can be assessed by the success rate in identifying the best decoy structure. We found that the performance is worse in identifying the best decoy structures based on statistical energies. This is a common challenge for many scoring functions, due to the difficulty in accurate ranking near-native structures that have similar scores (energies). In some CASP12 cases, the predicted structures could be far from the native model (such as in decoys of T0932 and T0933, see Fig. 5). Under such circumstances, none of the predicted models are close enough to the native structures, making it very difficult to select the best decoy structures (out of bad predicted models).

The parameters of the NEPRE algorithm were not further fine-tuned, except for the cutoff distance in the NEPRE-F. The probability distributions of relative locations between amino acids were converted to statistical potentials using the Boltzmann relation. Therefore, the NEPRE algorithm is mainly built on the position preferences of neighboring amino acids in protein structures. The performance of the algorithm indicates that the orientation is critical for amino acid packing in protein structures. In this study, both NEPRE-F (with cutoff = 6 Å) and NEPRE-R considered only the nearest neighbors. The distance dependency of the statistical potential function is not explicitly described. In principle, by extending the distance cutoff to larger values, longer-range interactions can be described using a similar approach. An immediate extension is to develop a multi-layer neighborhood preference-based energy function by dividing the neighbors into layers. We tested this multi-layer potential energy and found the performance was comparable the the NEPRE-F (with r_c_ = 6) by including the first two layers (r = 6 and r = 7). The inclusion of more layers deteriorated the performance in recognizing the native structures from the decoys (see Figure S9 and Table S2). The multi-layer implementation may need a weighting scheme and distance-dependent angular space gridding to improve the performance.

In summary, the neighborhood of amino acids in protein structures was statistically analyzed, and the discovered preferences were quantified using the types and relative positions of the neighboring amino acids. Based on the neighborhood preference, the program NEPRE was implemented to assess protein structure quality. The results showed that the NEPRE program can identify native (or near native) structures from decoy sets, providing a foundation for extended applications in protein structure assessment and prediction studies.

## Supplementary information

Supplementary Information.

## Data Availability

The source codes for NEPRE are available via https://github.com/LiuLab-CSRC/NePre.
