# Author Correction: Neighborhood Preference of Amino Acids in Protein Structures and its Applications in Protein Structure Assessment

**DOI:** 10.1038/s41598-020-69837-8

**Published:** 2020-07-28

**Authors:** Siyuan Liu, Xilun Xiang, Xiang Gao, Haiguang Liu

**Affiliations:** 1 Complex Systems Division, Beijing Computational Science Research Center, Haidian, Beijing, 100193 China; 2 School of Software Engineering, University of Science and Technology of China, Hefei, Anhui 230026 China; 3 Physics Department, Beijing Normal University, Haidian, Beijing, 100875 China

Correction to: *Scientific Reports* https://doi.org/10.1038/s41598-020-61205-w, published online 09 March 2020

This Article contains typographical errors in the Acknowledgements section.

“The authors are grateful to Prof. Yang Zhang and Dr. Chengxin Zhang from University of Michigan for the help with the decoys and the potential energy evaluations. This project is supported by National Natural Science Foundation of China (Nos. 11575021, U1530402, U1430237, 1181101028).”

should read:

“The authors are grateful to Prof. Yang Zhang and Dr. Chengxin Zhang from University of Michigan for the help with the decoys and the potential energy evaluations. This project is supported by National Natural Science Foundation of China (Nos. 11575021, U1930402, U1430237, 1181101028).”

