# Supplementary material for: Neighborhood Preference of Amino Acids in Protein Structures and its Applications in Protein Structure Assessment
Source: Sci Rep. 2020 Mar 9;10:4371. doi: 10.1038/s41598-020-61205-w (PMC7062742; doi:10.1038/s41598-020-61205-w)
Supplement: Supplementary file 1 — Supplementary Information. [file 41598_2020_61205_MOESM1_ESM.pdf]

Neighborhood Preference of Amino Acids in Protein Structures and its Applications in Protein Structure Assessment

Siyuan Liu[1,2], Xilun Xiang[1,2], Xiang Gao[1,2], Haiguang Liu[1,*]

[1] Complex Systems Division, Beijing Computational Science Research Center, Beijing, 100193, China

[2] School of Software Engineering, University of Science and Technology of China, Hefei, Anhui, 230026 China

Supplementary Information

This supplementary material contains the following information to support the main text:

1. The size distributions of 20 residues (Figure S1).

2. The angle parameter discretization at four levels (N=15,20,25,30):

- Figure S2 shows the performance on the Modeller dataset evaluation (using NEPRE-F with cutoff=6Å).
- Figure S3 shows the statistics of un-sampled sections.
- The performance in recognizing native structures is summarized in Table S1 for the four discretization schemes.

3. The structure assessment performance comparison with other methods. A representative decoy set **(1BYIA)** was used to show the correlation between scores(energies) and the RMSD values with respect to native structure (Figure S4).

4. The performance comparison on CASP12 dataset with other methods (Figure S5,S6,S7).

5. The comparison for intra-chain and inter-chain neighborhood preferences. The Jensen-Shannon divergence (D_{JS}) was used to measure the difference between the two distributions (intra- or inter- chain) for the neighboring case between any two amino acids.

Where D_{JS} is defined as:

$$D_{JS}(p||q) = \frac{1}{2}D_{KL}(p\left|\frac{p+q}{2}\right) + \frac{1}{2}D_{KL}(q||\frac{p+q}{2})$$

And D_{KL} is the Kullback-Leibler divergence:

$$D_{KL}(p||q) = \sum_{x} p(x)log\left(\frac{p(x)}{q(x)}\right)$$

Here, the p(x) and q(x) are the two distribution functions in the parameter space {x}.

Because D_{JS} is symmetric and bounded to [0,1] (for the base 2 logarithm), we used D_{JS} to measure the differences between the two distributions. The D_{JS} for 20x20 pairs of amino acids were summarized in Figure S8.

6. The decoy datasets:

- The simulation decoy datasets are available from Zhanglab at https://zhanglab.ccmb.med.umich.edu/decoys/
- The CASP12 decoy sets used in this study are uploaded to Github at: https://github.com/TangYuan-Liu/NEPRE_dataset_used

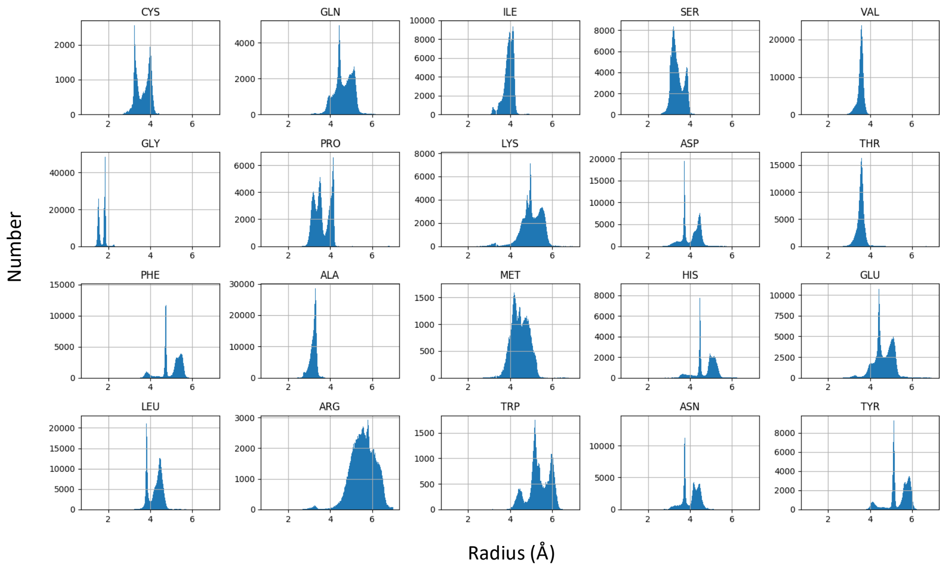

Figure S1. Distributions of amino acid radius. The statistics are based on the dataset composed of 14,647 high-resolution protein structures (BLAST $p<10^{-7}$). The radius (in Å) is defined as the largest distance between any atom and the geometry center of the amino acid. Each distribution is fitted using a Gaussian function, and the mean values are used as the characteristic radius for that amino acid.

Figure S2. The NEPRE-F performance on the Modeller dataset with four discretization schemes for the angle parameter space (next page). The θ [0,pi) and φ [0,2*pi] space was divided to 15x15, 20x20, 25x25, or 30x30 sections to describe the orientation dependent energy functions (see equation 4 in the main text). 20 decoy sets in the Modeller dataset were evaluated using each discretization scheme, and calculated energies were plotted against the RMSD values with respect to their native structures. The results suggest that the discretization scheme of 20x20 is sufficient for accurate assessment. The RMSD was in Å, and the energy is in the unit of *k*T.

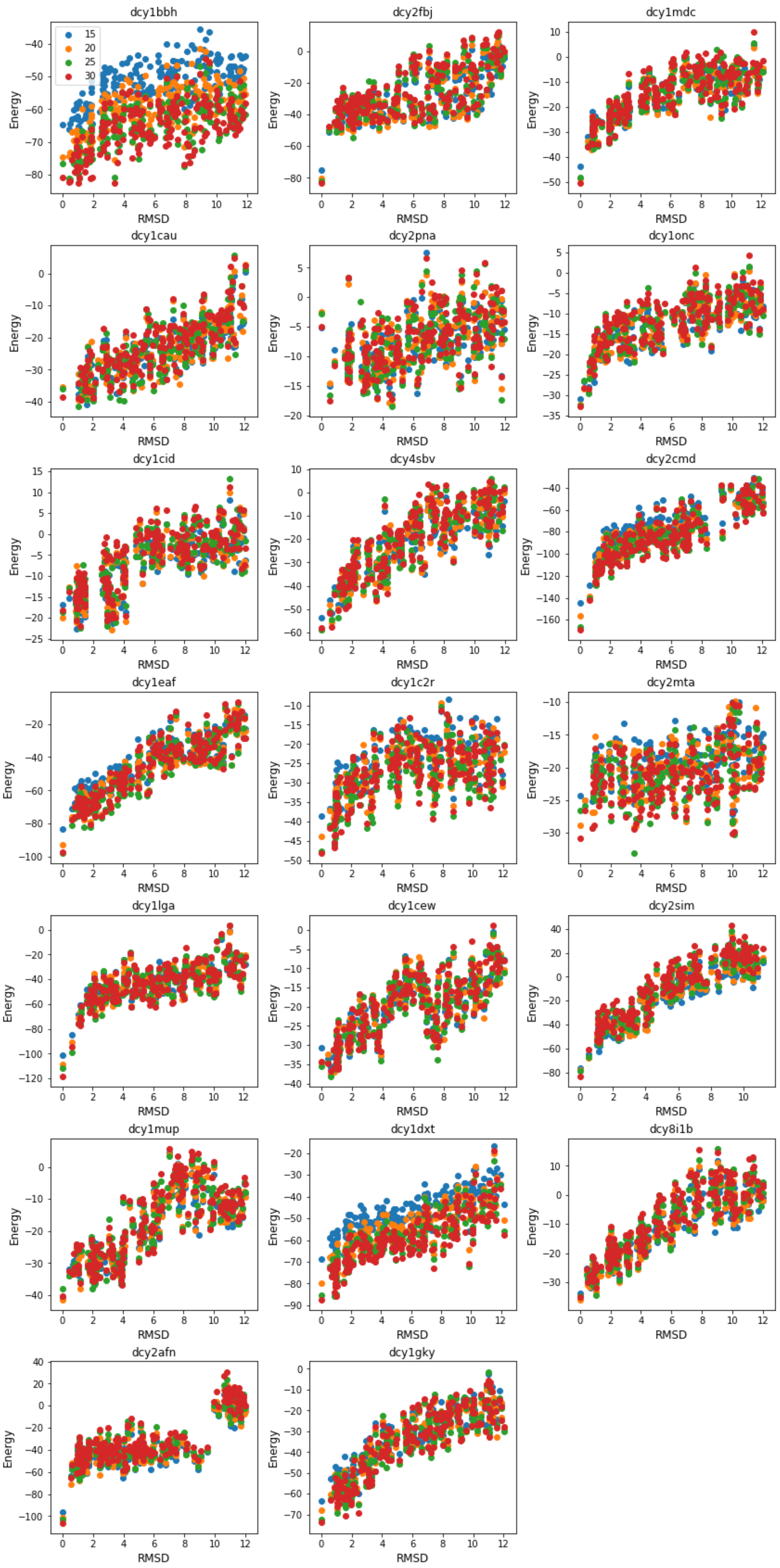
dcy1bbh
15
20
25
30
dcy2fbj
dcy1mdc
dcy1cau
dcy2pna
dcy1onc
dcy1cid
dcy4sbv
dcy2cmd
dcy1eaf
dcy1c2r
dcy2mta
dcy1lga
dcy1cew
dcy2sim
dcy1mup
dcy1dxt
dcy8i1b
dcy2afn
dcy1gky
Energy
RMSD

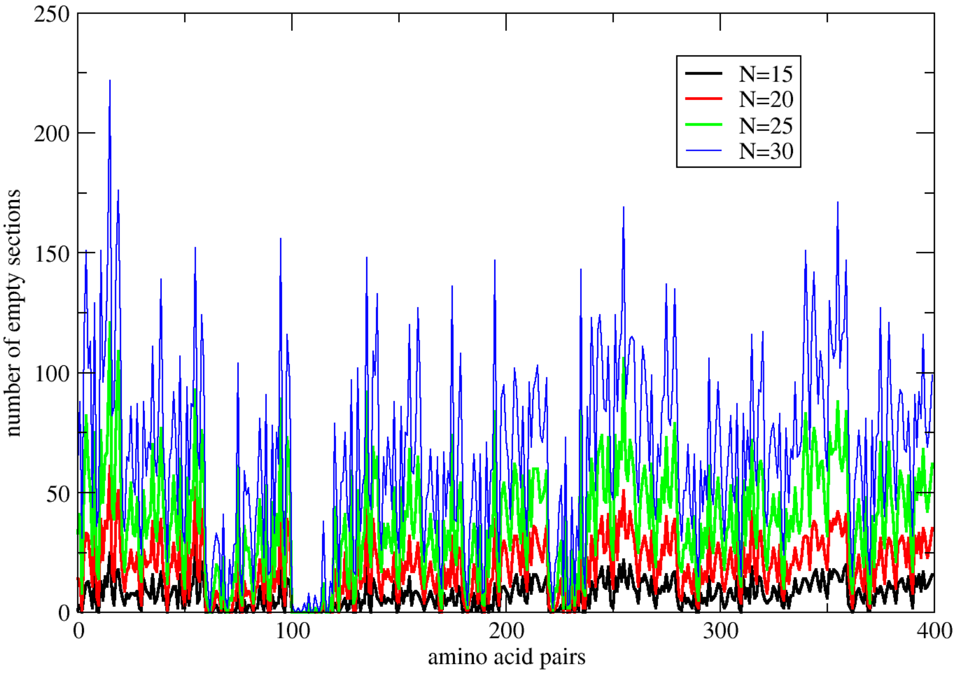

Figure S3. The statistics of un-sampled sections for four discretization schemes. The number of un-sampled sections for all pairs of amino acids. Finer discretization resulted more un-sampled sections. With N=20 (400 sections), the un-sampled sections are fewer than 18.8 on average (red curve) for all amino acid pairs. In terms of percentage, the un-sampled regions are 3.2%, 4.6%, 6.0%, 7.3% for N=15,20,25,30. We choose a discretization scheme with N=20 to balance the function accuracy and statistical significance. For un-sampled sections, the probability is 0, corresponding to infinite high energy according to Boltzmann relation. To avoid such singularities, bi-linear interpolation in the potential energy space was carried out for those regions.

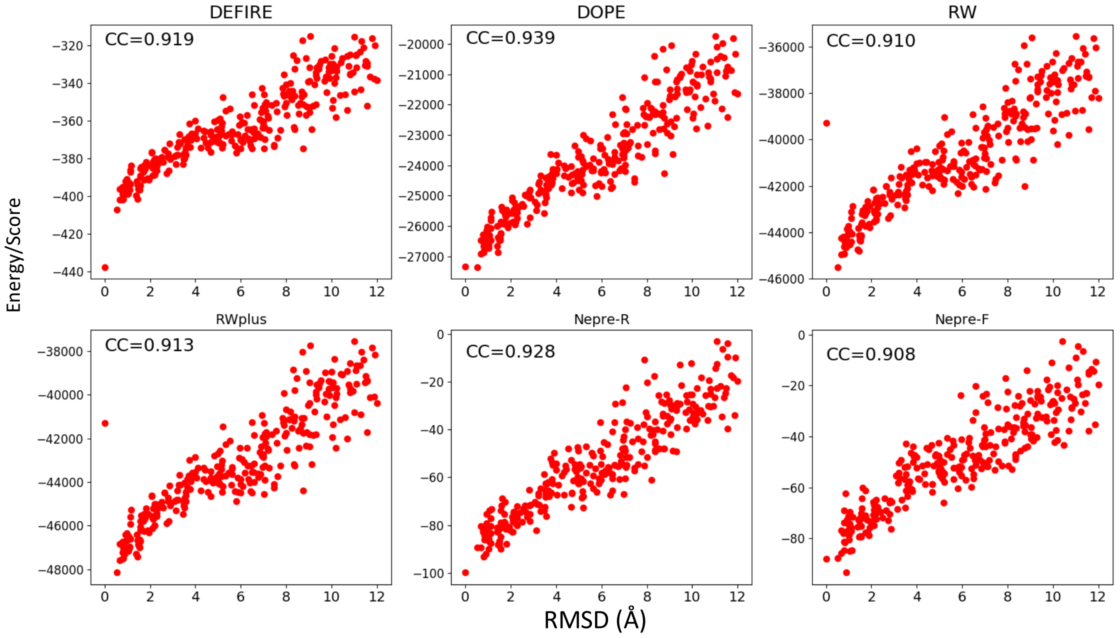

Figure S4. The correlation between energy function and the structure difference compared to native state (measured using RMSD) for decoy set 1BYIA in I-TASSER(b). The energies calculated using these 6 methods have strong positive correlations to the structure qualities (RMSD values).

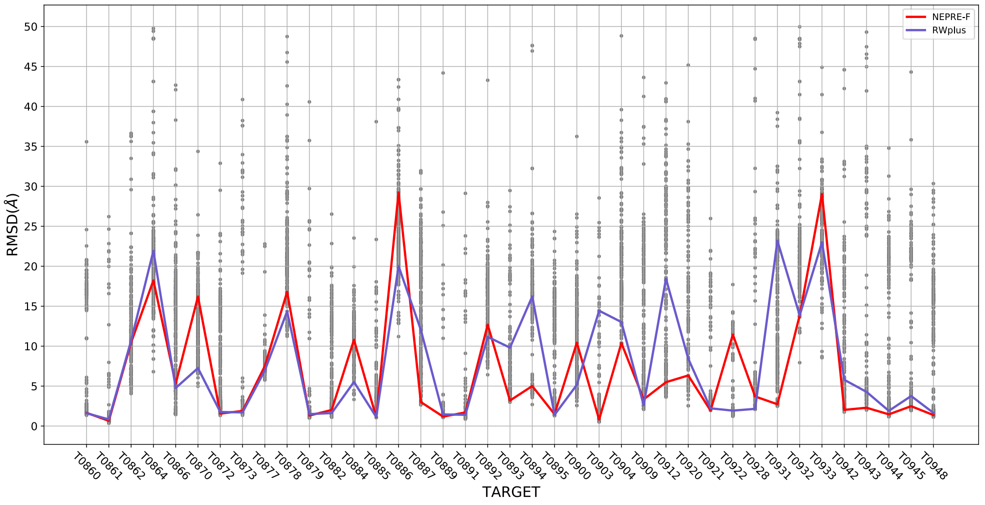

Figure S5. The performance of NEPRE-F compared to RWplus on the CASP12 dataset. The dots are the distributions of RMSD of decoy structures with respect to their native structure. The lines indicate the identified structures with the lowest energies.

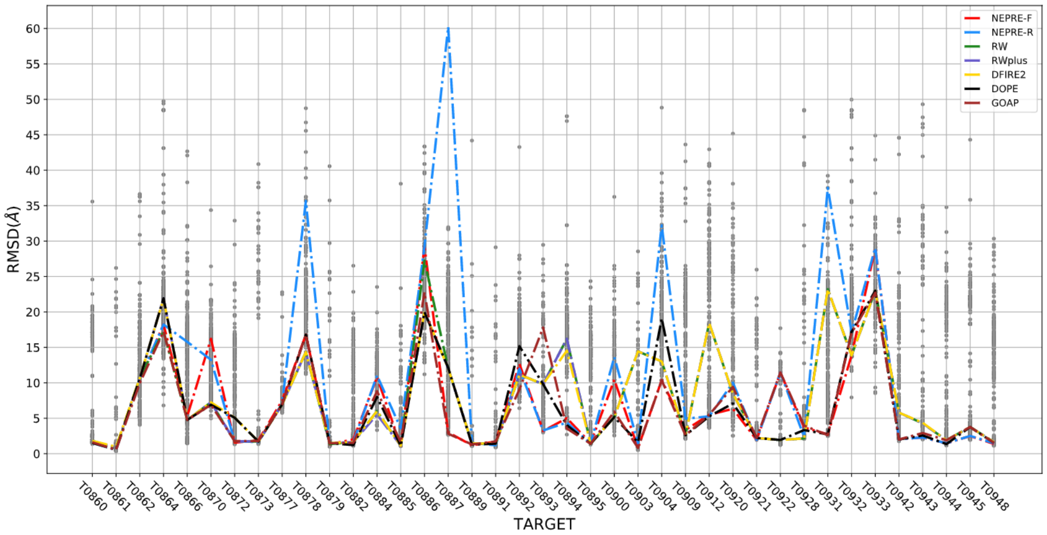

Figure S6. The performance comparison using CASP12 dataset for six methods. The colored lines show the selected model with the lowest energies. The RMSD was calculated with respect to the native structure solved using crystallography method.

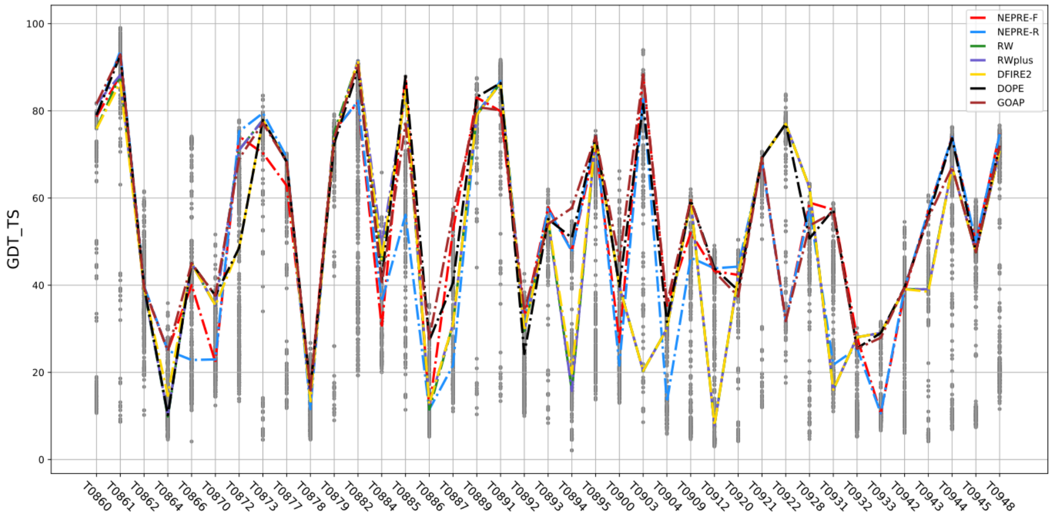

Figure S7. The performance comparison of six methods. The plot scheme is the same as Figure S6, except that the RMSD is replaced with the GDT_TS scores, which evaluate the similarity to the native structure..

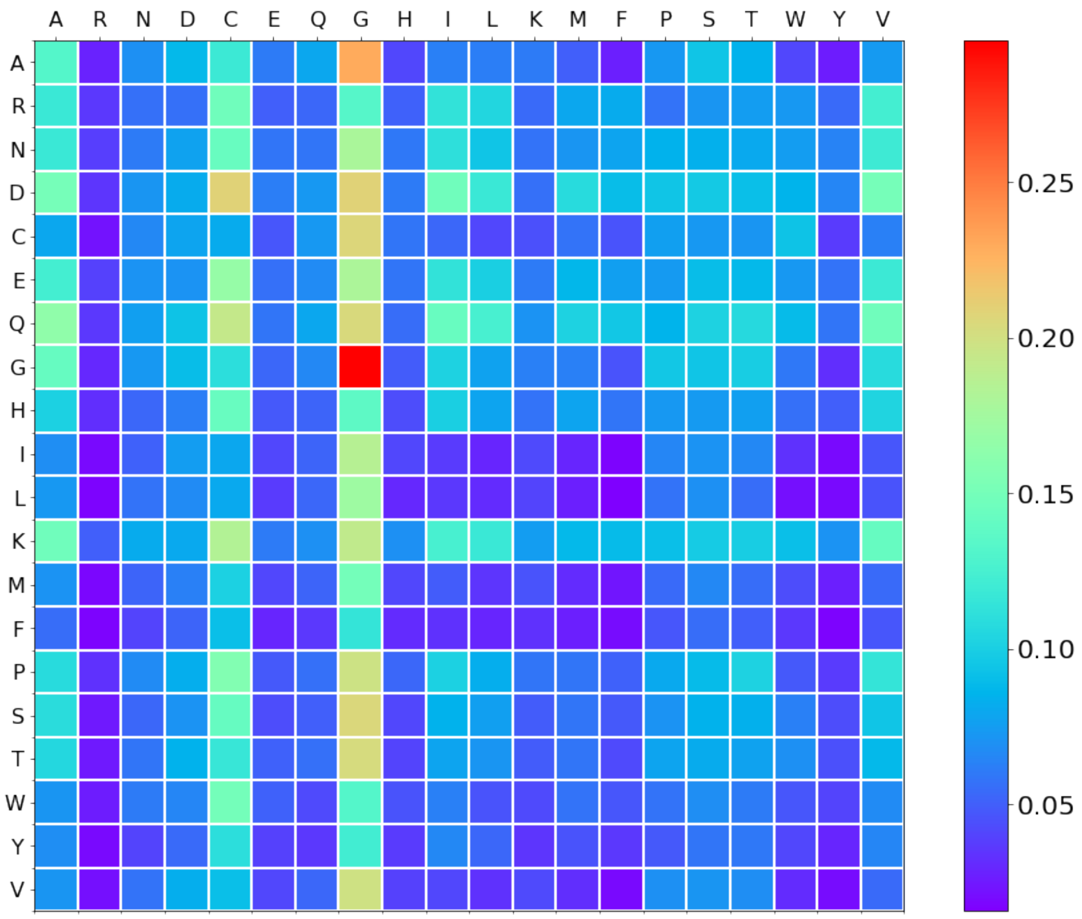

Figure S8. The comparison of neighborhood preferences derived from single chain protein structures and that from protein complex interfaces. The Jensen-Shannon Divergence was used to measure the difference between the two distributions for each pair of amino acids (in the unit of bit, since the *base 2 logarithm* was used in the calculation). Each row summaries the neighborhoods centered at a particular amino acid type; and each entry in a row corresponds to a neighboring amino acid distributed around the centered amino acid. The columns for alanine, cysteine, glycine and valine reveal large differences in the neighborhood preferences; the divergences are small in other cases, indicating similar preferences.

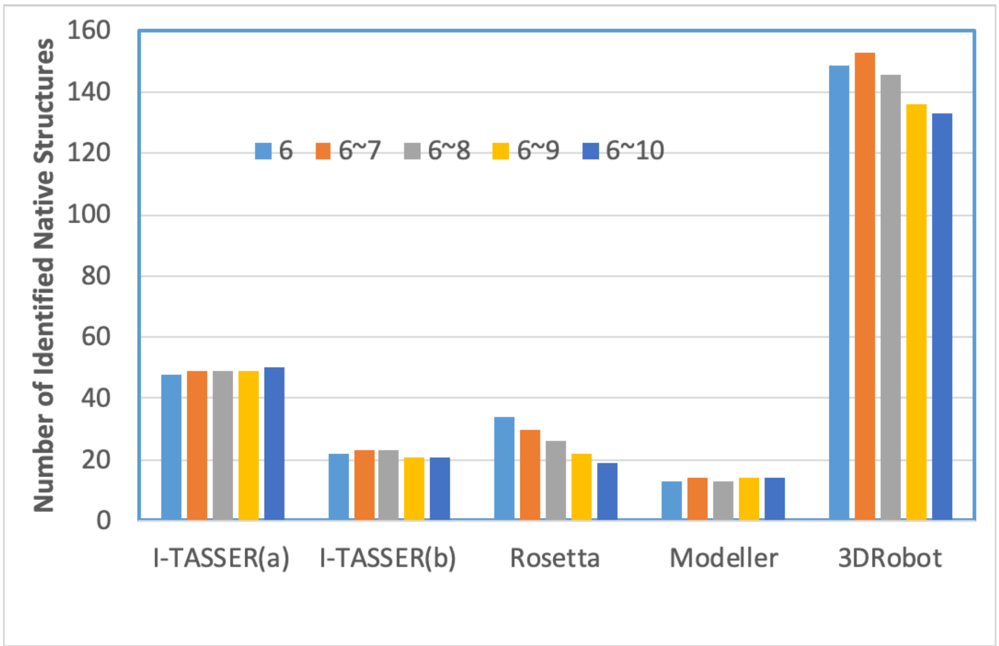

Figure S9. The performance of multilayer NEPRE in identifying native structures. The layer information (the numbers indicate the cutoff distances, in Å) is shown in legends. Each column shows the number of identified native structures.

Table S1. The numbers of identified native structures using four discretization schemes for angle parameter space.

Number of Grids(N)		15	20	25	30
Modeller	Top1	13	13	13	15
(20 decoy sets)	Top5	14	16	15	16
	Top10	15	18	17	19

The results presented in the main text was obtained with N=20.

Table S2. The performance of NEPRE with multilayer potential energies.

Layer (6,7)[#]					
	**3DRobot**	**I-TASSER(a)**	**I-TASSER(b)**	**Rosetta**	**Modeller**
**top1**	153	49	23	30	14
**top5**	177	49	32	50	16
**top10**	183	49	36	52	16
**Layer (6, 7, 8)**					
	**3DRobot**	**I-TASSER(a)**	**I-TASSER(b)**	**Rosetta**	**Modeller**
**top1**	146	49	23	26	13
**top5**	174	49	34	50	15
**top10**	183	49	38	52	15
**Layer (6, 7, 8, 9)**					
	**3DRobot**	**I-TASSER(a)**	**I-TASSER(b)**	**Rosetta**	**Modeller**
**top1**	136	49	21	22	14
**top5**	164	49	30	49	16
**top10**	175	49	36	53	16
**Layer(6, 7, 8, 9, 10)**					
	**3DRobot**	**I-TASSER(a)**	**I-TASSER(b)**	**Rosetta**	**Modeller**
**top1**	133	50	21	19	14
**top5**	158	50	30	47	17
**top10**	169	50	37	50	17

[#] The decoy models were ranked using the total energy: $E_{tot} = \sum_{i\in\{layer\}} E_i$ where E_i is the potential energy function derived from the layer i. The other fields are the same as Table 4 in the main text.
